# Effect of Chloroform Application on Roughness and Wettability of the Root Canal Walls in Endodontic Retreatment 

**DOI:** 10.30476/DENTJODS.2021.88414.1329

**Published:** 2022-09

**Authors:** Davoud Jamshidi, Mohammadreza Tahriri, Heydar Mosleh, Mohammad Madadpour, Soolmaz Heidari, Mahmood Alipour Heydari, Mohamad Javad Kharazifard

**Affiliations:** 1 Dept. of Endodontics, Dental Caries Prevention Research Center, Qazvin University of Medical Sciences, Qazvin, Iran; 2 Postdoctoral Fellow Researcher, Marquette University School of Dentistry, Milwaukee, WI 53233, USA; 3 General Dentist, Student Research Committee, Qazvin University of Medical Sciences, Qazvin, Iran; 4 Dept. of Operative Dentistry, Dental Caries Prevention Research Center, Qazvin University of Medical Sciences, Qazvin, Iran; 5 Dept. of Biostatistics, Qazvin University of Medical Sciences, Qazvin, Iran; 6 Dental Research Center, Dentistry Research Institute, Tehran University of Medical Sciences, Tehran, Iran

**Keywords:** Sealer, Chloroform, Gutta percha, Dentin, Contact angle

## Abstract

**Statement of the Problem:**

The success of root canal therapy depends on root canal irrigation, disinfection, and sealing of root canal. Wettability and roughness of root dentine surface are important factors in root canal disinfection and sealing.

**Purpose:**

This study aimed to assess the effect of chloroform application on roughness and wettability of the root canal walls in endodontic retreatment.

**Materials and Method:**

This *in vitro* experimental study evaluated 70 sound extracted human anterior teeth. The specimens were then randomly assigned to 7 groups as follows: Chloroform (group 1), 1g gutta percha+chloroform (group 2), 2g gutta percha+ chloroform (group 3), 1g gutta percha+ 1g sealer+ chloroform (group 4), 2g gutta perch+ 2g sealer+chloroform (group 5), 1g sealer + chloroform (group 6) and 2g sealer + chloroform (group 7). One drop of distilled water was placed on each tooth to measure the contact angle and wettability. Photographs were obtained of an area measuring 50×50 µm^2^ in three directions under an atomic force microscope to measure the roughness. The tooth blocks were exposed to the abovementioned mixtures for 10 min, and then rinsed with saline. The roughness and wettability of each sample were measured before and after treatment. Data was analyzed using one-way ANOVA and Tukey’s test.

**Results:**

The roughness of all groups significantly decreased following treatment, except for groups 1 and 4. The contact angle increased in all groups after treatment (except for the chloroform group), which indicated decreased wettability. The roughness and the contact angle have shown no correlation.

**Conclusion:**

Application of chloroform for removal of gutta-percha and sealer in endodontic retreatment decreases the roughness and wettability of dentine.

## Introduction

One major advantage of using gutta-percha for root canal obturation is its relatively easy retrieval [ 1 ]. Several techniques have been suggested for gutta-percha retrieval, such as the use of rotary files and ultrasonic devices, heat, manual files with heat, or application of a solvent [ 2 ]. Chloroform is a solvent commonly used for removal of root filling materials. It is the fastest and most efficient solvent for softening of gutta-percha. Other solvents have also been tested but have shown lower efficacy for this purpose [ 3 ].

The mechanical status of the root canal wall and its degree of smoothness/roughness highly depend on its mineral content, the residual organic material, and the preparation technique [ 4 - 5 ]. In endodontic retreatment, optimal adaptation of sealer to the root canal wall is an important parameter in achieving a hermetic seal [ 6 ]. 

One important property for sealers is their adhesion ability to the root canal walls. This property depends on the wettability of the root canal wall by the sealer, which affects its adaptation to root dentin [ 7 - 8 ]. The contact angle is a beneficial index for assessment of the wettability of a liquid. It refers to the angle formed between a liquid and a solid surface. An inverse correlation exists between the wettability and contact angle [ 9 - 10 ]. Wettability is influenced by three factors: namely the free energy of the solid surface, the surface topography of the material and its roughness, and the liquid viscosity [ 11 - 13 ]. Wetting of the dentin surface with low energy is more difficult than the enamel surface. By increasing the surface energy of enamel and dentin, their wettability increases and subsequently, the adhesion is enhanced [ 14 ]. Dentin wettability highly depends on the chemical irrigating solutions used, surface roughness, and its level of hydration. It is also affected by the density of dentinal tubules [ 15 ]. Moreover, presence of the smear layer in the root canal system affects the wettability [ 16 ]. The correlation between roughness and wettability of surface is described by the Wenzel equation [ 17 ]. 

It seems that dentin surface wettability might influence the root canal sealing and disinfection [ 4 - 8 , 15 ]. Furthermore, the studies on the effect of chloroform, used for gutta-percha and sealer removal, on roughness and wettability are limited. Therefore, this study was designed to assess the effect of chloroform, gutta-percha, and sealer on roughness and wettability of the root canal walls. 

## Materials and Method

This *in vitro* experimental study evaluated sound extracted human anterior teeth. The study was approved by the Ethics Committee of Qazvin University of Medical Sciences (Code: IR.QUMS.REC.1394.650). Sample size was calculated to be 70.

The inclusion criteria were defined as the teeth free from root caries, fractures, or cracks to be enrolled in the study. The coronal and apical thirds of the roots were cut by a high-speed hand-piece. The remaining middle third was longitudinally sectioned into two halves using a diamond bur. Tooth blocks were mounted in auto-polymerizing acrylic resin and were then polished by 600- grit abrasive paper (Matador, Germany). The smear layer was removed by 17% ethylenediaminetetraacetic acid (EDTA) (Sigma-Aldrich, St. Louis, MO, USA) and 5.25% sodium hypochlorite (NaOCl) (Sigma-Aldrich, St. Louis, MO, USA). Each irrigant was applied on dentin for 1 minute. 

The specimens were then randomly assigned to seven groups including Group 1: 10 cc chloroform, Group 2: 1g gutta percha/10cc chloroform, Group 3: 2g gutta percha /10cc chloroform, Group 4: 1g gutta percha/1g sealer/ 10cc chloroform, Group 5: 2g gutta perch/2g sealer/10cc chloroform, Group 6: 1g sealer/ 10cc chloroform, and Group 7: 2g sealer/10cc chloroform.

 One drop of distilled water was placed on each tooth by a micro-syringe; they were photographed using a digital camera with a close-up lens (Sony, Tokyo, Japan). To measure the roughness value (Ra) of each specimen, photographs were obtained of an area measuring 50×50 µm^2^ in three directions under an atomic force microscope (Dualscope/Rasterscope C26, DME, Herlev, Denmark). In Group 1, 10 cc of chloroform was used alone. In groups with gutta-percha, 1 and 2 g of gutta-percha (Gapadent Co., Ltd, TianJin City, China) were separately weighed using a digital scale (GF-300; A&D company Ltd., Tokyo, Japan) and separately dissolved in 10 cc of chloroform (Golchai Co, Alborz, Karaj, Iran). In groups containing sealer, AH26 sealer (Dentsply DeTrey GmbH, Konstanz, Germany) was prepared according to the manufacturer’s instructions. Set form of the sealer, in amounts of 1 and 2g, was separately added to 10cc of chloroform. In group 4, 1g gutta-percha and 1g sealer and in group 5, 2g gutta-percha and 2g sealer were added to 10 cc of chloroform. The tooth blocks were exposed to the abovementioned mixtures for 10 min, and were then rinsed with saline to ensure no gutta-percha remained on dentin surface. If the surface was contaminated with gutta-percha, it was washed with chloroform and then saline. The roughness and wettability of the specimens were then measured again. For measurement of wettability, the contact angle of each drop of water relative to the root dentin surface was measured on each image before and after rinsing using the ruler computer software. Using V614r1 software (Veeco, Santa Barbara, CA, USA), the Ra value was measured in an area measuring 50×50µm^2^. The mean and standard deviation values of roughness and wettability (contact angle) were calculated in each group and compared using one-way ANOVA and Tukey’s post-hoc test (*p*< 0.05). The correlation of roughness and wettability was analyzed using Pearson correlation analysis.

## Results

The roughness value significantly decreased in all groups following treatment except for Groups 1 and 4, in which the reduction was not significant (Table 1). The increase in the contact angle, which indicated the decrease in wettability, was significant in some groups (Table 2). Only the chloroform group (Group 1) showed significant reduction of contact angle. Statistical analysis revealed no significant correlation between roughness and contact angle. The roughness of groups was not significantly different before treatment. After treatment, Groups 1, 2 and 4 showed significant differences in roughness values with Groups 3 and 5. The contact angle of groups was not significantly different before treatment. After treatment, Group 1 demonstrated significant differences with all groups except for Groups 5 and 6. A significant difference was noted in this respect between Group 4 and Group 6. Increasing the amount of gutta-percha in chloroform from 1g to 2g, (groups 2 and 3) significantly decreased the wettability. The same result was noted by increasing the amount of sealer from 1g to 2g in chloroform (groups 6 and 7). No such a trend was observed in groups containing chloroform, gutta-percha, and sealer. 

**Table 1:** Mean and standard deviations of roughness values of root dentine surface before and after treatment and *p* value

Roughness(µm)	Group 1	Group 2	Group 3	Group 4	Group 5	Group 6	Group 7
Mean±SD							
Before	116.87±19.1	161.98±66.4	116.85±24	135.92±58.2	137.78±41.1	136.60±19.7	145.60±15.8
After	107.34±18.4	107.41±26.3	75.88±13.4	105.06±31.5	75.70±25.4	98.88±14.3	88.72±11.8
*p* value	0.261	0.047	0.004	0.22	0.008	0.003	0.001

**Table 2:** Mean and standard deviation of contact angles of root dentine surface before and after treatment and *p* value

Contact Angle (degrees)	Group 1	Group 2	Group 3	Group 4	Group 5	Group 6	Group 7
Mean±SD							
Before	74.08±9.3	72.62±10.7	67.88±5.3	69.99±6.8	68.84±6.6	65.69±8.7	66.07±9.2
After	63.69±8.9	78.25±10.5	75.19±5.7	83.80±8.4	73.83±7.6	72.40±5.2	76.63±9.2
*p* value	0.005	0.171	0.008	0.001	0.18	0.069	0.019

## Discussion

This study assesses the effect of chloroform alone and in combination with gutta-percha and sealer on wettability and roughness of root canal walls *in vitro*. In this study, the baseline wettability and roughness of specimens were measured before exposure to the materials, because each dentin specimen might structurally be different from others, or the quality of specimen preparation may vary. In addition, the differences in dentin structure may explain the reason why roughness or wettability in some groups showed significant differences before and after treatment, while these differences were not significant in some other groups. However, by adopting this methodology, each specimen served as its own control. Also, the specimens were standardized as much as possible, and each dentin specimen was randomly chosen among the prepared specimens.

Two different amounts of sealer and gutta-percha were evaluated in this study because there was no consensus or previous study on the minimum amount of chloroform required for dissolving each 1g of gutta-percha or sealer. Although 10 cc of chloroform is more than the amount used in the clinical treatments, it is required for adequate treatment of dentin blocks and a complete softening of gutta percha.

Since chloroform dissolves lipids, it may alter the chemical properties of dentin surface [ 18 ]. The current results indicate that application of chloroform decreases the roughness value, but not significantly. It also decreases the contact angle and, consequently, increases the wettability; this increase is statistically significant.

In a study by Erdemir *et al.* [ 18 ], the amount of magnesium decreased following the application of chloroform. Nonetheless, no significant reduction was noted in microhardness. Kufman *et al.* [ 19 ], observed that the calcium and phosphorous contents of human dentin changed following the application of chloroform; although this change was not statistically significant. Rostein *et al.* [ 20 ], indicated that chloroform decreased the enamel and dentin microhardness. The microhardness test can indirectly determine the reduction in mineral content of tooth structure. Differences in the results of studies can be due to variations in study design, type of teeth, use of different tooth parts (root dentin or coronal dentin), age of teeth, method of application of material, its duration of application, and the composition of material tested [ 18 - 20 ].

Jain *et al.* [ 21 ], indicated that application of chloroform softened the gutta-percha, which was then condensed into the dentinal tubules. This can compromise root canal irrigation and subsequent elimination of microorganisms from the root canal system. The roughness value increases when the tubules are wide open. The roughness value is correlated with the availability of patent dentinal tubules. Evidence shows that smear layer removal can lead to opening of dentinal tubules and subsequently increase the roughness [ 15 ]. The reduction in roughness value, although insignificant in some groups, may be due to the obstruction of dentinal tubules by the softened and condensed gutta-percha. Nonetheless, evidence shows that application of chloroform may rarely result in complete obstruction of all dentinal tubule cross-sections by the residual root filling materials [ 22 ]. 

Use of chloroform-along with gutta-percha or sealer or both- in root canal treatment decreases the wettability of dentin. The results show that increasing the amount of sealer and gutta-percha separately from 1g to 2g further decrease the wettability. However, this trend is not observed in gutta-percha plus sealer groups. 

It may be concluded that in endodontic retreatment by use of chloroform, the amount of sealer and gutta-percha present in the root canal system can affect the wettability of dentin. This wettability plays an important role in reobturation of the canal, in terms of equal distribution of sealer and its optimal adhesion to the canal walls. In our study, the use of any material in endodontic retreatment decreased the roughness in all groups. The reduction in roughness of a wettable surface is associated with decreased wettability, according to the Wenzel equation. This trend was observed in all groups in our study (although not significant in some groups) except for chloroform group. Evidence shows that in a wettable surface, roughness can increase wettability by increasing the surface area. However, excessive increase in roughness can prevent the liquid flow and lead to entrapment of air bubbles [ 23 ]. 

The contact angle is the result of the solid-liquid surface energy balance. The penetration coefficient is related to the surface tension and viscosity of liquid [ 13 ]. Water was used for measuring the contact angle in all specimens in this study. The influential factors on the contact angle that are related to the solid surface include the hydrophobic effect due to the altered porosity and the resultant roughness value. Also, the surface free energy generated following changes in 3D molecular structure of the solid surface plays a critical role in wettability. By measuring the contact angle to determine the wettability of a solid surface, it would be difficult to find out whether roughness or changes in the solid surface energy have a greater effect on wettability [ 7 ].

Obstruction of dentinal tubules and subsequently decreased roughness and wettability- can impair the process of root canal disinfection and achieving an optimal seal [ 24 ]. By increasing the wettability of solutions on dentine surface, the irrigant can penetrate into the uninstrumented area of root canal system and improves antimicrobial activity [ 25 ]. Thus, in order to improve the quality of endodontic retreatment and increase its success rate, it is suggested to maintain the dentinal tubules open and increase the roughness and wettability of dentin. In addition, reports show that NaOCl, EDTA, H2O2, and calcium hydroxide can change the wettability of dentine [ 15 , 26 - 27 ]. Future studies are required to further scrutinize this topic and take into account a higher number of confounding factors.

## Conclusion

The current results indicate that use of chloroform for removal of gutta-percha and sealer in endodontic retreatment decreases the roughness and wettability of dentin.

## Conflict of Interest

The authors declare that they have no conflict of interest.

## References

[1] Johnson W, Kulild JC, Tay F, Hargreaves KM, Berman LH (2016). The core science of endod-ontics, obturation of the cleaned and shaped root canal system. Cohen’s Pathways of the Pulp.

[2] Good ML, McCammon A ( 2012). A removal of gutta-percha and root canal sealer: a literature review and an audit comparing current practice in dental schools. Dent Update.

[3] Mushtaq M, Farooq R, Ibrahim M, Khan FY ( 2012). Dissolving efficacy of different organic solvents on gutta-percha and resilon root canal obturating materials at different immersion time intervals. J Conserv Dent.

[4] Sakhaei Manesh V, Giacomin P, Stoll R ( 2017). Quantitative evaluation of root canal surface roughness after filing with adaptive reciprocating and continuous rotary instruments. Microsc Res Tech.

[5] Patil CR, Uppin V ( 2011). Effect of endodontic irrigating soluteons on the microhardness and roughness of root canal dentin: an in vitro study. Indian J Dent Res.

[6] Huang Y, Orhan K, Celikten B, Orhan AI, Tufenkci P, Sevimay S ( 2018). Evaluation of the sealing ability of different root canal sealers: a combined SEM and micro-CT study. J Appl Oral Sci.

[7] Tummala M, Chandrasekhar V, Rashmi AS, Kundabala M, Ballal V ( 2012). Assessment of the wetting behavior of three different root canal sealers on root canal dentin. J Conserv Dent.

[8] Ha JH, Kim HC, Kim YK, Kwon TY ( 2018). An Evaluation of Wetting and Adhesion of Three Bioceramic Root Canal Sealers to Intraradicular Human Dentin. Materials (Basel).

[9] Guo L, Wong PL, Guo F ( 2015). Correlation of contact angle hysteresis and hydrodynamic lubrication. Tribol Lett.

[10] Kontakiotis EG, Tzanetakis GN, Loizides AL ( 2007). A comparative study of contact angles of four different root canal sealers. J Endod.

[11] Farge P, Alderete L, Ramosb S ( 2010). Dentin wetting by three adhesive systems: influence of etching time, temperature and relative humidity. J Dent.

[12] Pantoja CAMS, Silva DHD, Soares AJ, Ferraz CCR, Gomes BP, Zaia AA, et al ( 2018). Influence of ethanol on dentin roughness, surface free energy and interaction between AH Plus and root dentin. Braz Oral Res.

[13] Van Noort R, Barbour M, Van Noort R (2013). Basic science for dental materials. Introduction to Dental Materials..

[14] Sofan E, Sofan A, Palaia G, Tenore G, Romeo U, Migliau G ( 2017). Classification review of dental adhesive systems: from the IV generation to the universal type. Ann Stomatol (Roma).

[15] Hu X, Ling J, Gao Y ( 2010). Effects of irrigation solutions on dentin wettability and roughness. J Endod.

[16] Aguilar-Mendoza JA, Rosales-Leal JI, Rodríguez-Valverde MA, González-López S, Cabrerizo-Vílchez MA (2008 ). Wettability and bonding of self-etching dental adhesives Influence of the smear layer. Dent Mater.

[17] Mohan RP, Vivekananda Pai AR ( 2015). The comparison between two irrigation regimens on the dentine wettability for an epoxy resin based sealer by measuring its contact angle formed to the irrigated dentine. J Conserv Dent.

[18] Erdemir A, Eldeniz AU, Belli S ( 2004). Effect of the gutta-percha solvents on the microhardness and the roughness of human root dentine. J Oral Rehabil.

[19] Kaufman D, Mor C, Stabholz A, Rotstein I ( 1997). Effect of gutta-percha solvents on calcium and phosphorus levels of cut human dentin. J Endod.

[20] Rotstein I, Cohenca N, Teperovich E, Moshonov J, Mor C, Roman I, et al ( 1999). Effect of chloroform, xylene, and halothane on enamel and dentin microhardness of human teeth. Oral Surg Oral Med Oral Pathol Oral Radiol Endod.

[21] Jain M, Singhal A, Gurtu A, Vinayak V ( 2015). Influence of ultrasonic irrigation and chloroform on cleanliness of dentinal tubules during endodontic retreatment: an in vitro SEM study. J Clin Diagn Res.

[22] Horvath SD, Altenburger MJ, Naumann M, Wolkewitz M, Schirrmeister JF ( 2009). Cleanliness of dentinal tubules following gutta-percha removal with and without solvents: a scanning electron microscopic study. Int Endod J.

[23] Ayad MF, Johnston WM, Rosenstiel SF (2009). Influence of dental rotary instruments on the roughness and wettability of human dentin surfaces. J Prosthet Dent.

[24] Viapiana R, Moinzadeh AT, Camilleri L, Wesselink PR, Tanomaru Filho M, Camilleri J ( Int Endod J 2016). Porosity and sealing ability of root fillings with gutta-percha and BioRoot RCS or AH Plus sealers. Evaluation by three ex vivo methods.

[25] Giardino L, Ambu E, Becce C, Rimondini L, Morra M ( 2006). Surface tension comparison of four common root canal irrigants and two new irrigants containing antibiotic. J Endod.

[26] Xu J, He J, Shen Y, Zhou X, Huang D, Gao Y, et al ( 2019). Influence of endodontic procedure on the adherence of enterococcus faecalis. J Endod.

[27] Yassen GH, Sabrah AH, Eckert GJ, Platt JA ( 2015). Effect of different endodontic regeneration protocols on wettability, roughness, and chemical composition of surface dentin. J Endod.

